# Evaluation of a Proposed Biodegradable ^188^Re Source for Brachytherapy Application: A Review of Dosimetric Parameters

**DOI:** 10.1097/MD.0000000000001098

**Published:** 2015-07-17

**Authors:** Abdollah Khorshidi, Marjan Ahmadinejad, S. Hamed Hosseini

**Affiliations:** From the Department of Physics, Parand Branch (AK, MA); Department of Biomedical Radiation Engineering, Science and Research Branch, Islamic Azad University, Tehran, Iran (SHH).

## Abstract

This study aimed to evaluate dosimetric characteristics based on Monte Carlo (MC) simulations for a proposed beta emitter bioglass ^188^Re seed for internal radiotherapy applications.

The bioactive glass seed has been developed using the sol-gel technique. The simulations were performed for the seed using MC radiation transport code to investigate the dosimetric factors recommended by the AAPM Task Group 60 (TG-60).

Dose distributions due to the beta and photon radiation were predicted at different radial distances surrounding the source. The dose rate in water at the reference point was calculated to be 7.43 ± 0.5 cGy/h/μCi. The dosimetric factors consisting of the reference point dose rate, *D*(*r*_0_,*θ*_0_), the radial dose function, *g*(*r*), the 2-dimensional anisotropy function, *F*(*r*,*θ*), the 1-dimensional anisotropy function, φ_an_^(r)^, and the *R*90 quantity were estimated and compared with several available beta-emitting sources.

The element ^188^Re incorporated in bioactive glasses produced by the sol-gel technique provides a suitable solution for producing new materials for seed implants applied to brachytherapy applications in prostate and liver cancers treatment. Dose distribution of ^188^Re seed was greater isotropic than other commercially attainable encapsulated seeds, since it has no end weld to attenuate radiation.

The beta radiation-emitting ^188^Re source provides high doses of local radiation to the tumor tissue and the short range of the beta particles limit damage to the adjacent normal tissue.

## INTRODUCTION

Over the last few decades, worldwide interest in the treatment of cancer has continually increased. Liver cancer is the sixth most common cancer worldwide.^1,2^ An estimated 30,640 new cases of liver cancer were expected to occur in the United States during 2013. More than 80% of these cases were rapid-growing hepatocellular carcinoma (HCC) tumors.^3^ The prognosis of HCC remains extremely poor, and a curative treatment (liver transplantation, surgical resection, and radiofrequency ablation) can only be carried out in approximately 25% to 30% of cases.^4^ Although surgery (hepatectomy or liver transplantation) is the main form of curative treatment, the majority of patients are not eligible for surgery due to extent of tumor and dysfunction of liver. Radiotherapy is a method of cancer treatment in which radiation is used to destroy a tumor, or, at least, to hinder its growth. The use of conventional external beam radiation therapy in HCC treatment has been limited by the low radiation tolerance of the cirrhotic liver that often resulted in radiation-induced liver disease (RILD). Internal radioisotope therapy is another technique that has been developed and used for the treatment of HCC. A variety of radioisotopes, such as Iodine-131, Yttrium-90, Rhenium-188, Holmium-166 etc, are applied for this purpose**.**^5,6^ Brachytherapy is a type of internal radiotherapy that places solid radioactive sources in or adjacent to target tissues. In this study, a beta-emitting ^188^Re biodegradable glass seed is proposed for the brachytherapy treatment of small hepatocellular carcinomas. Diverse photon- or electron-emitting radioactive isotopes have been utilized or suggested for use over brachytherapy. Beta emitter radionuclides such as ^32^P, ^188^Re, and ^90^Sr/^90^Y are the most commonly used sources in intravascular brachytherapy.^7–10^ The general properties of some beta emitter radionuclides with therapeutic potential are demonstrated in Table 1.^11,12^

**TABLE 1:**
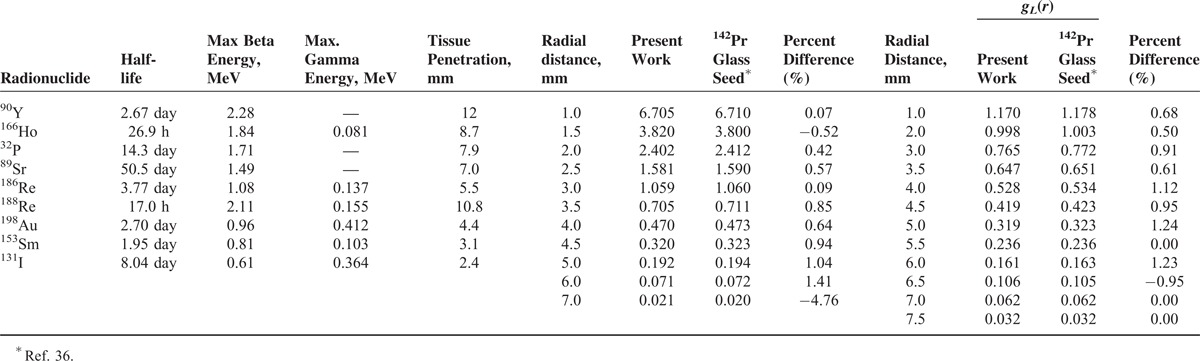
Several Beta Emitter Radionuclides With Therapeutic Potential for Brachytherapy (Left); Comparison of the Monte Carlo Calculated Dose Rate (cGy/h/μCi) Amounts of the ^142^Pr Glass Source With the Published Data (Middle); Comparison of the Monte Carlo Calculated Radial Dose Function of the^142^Pr Glass Source With the Published Data (Right)

^188^Re (17 h half-life, 84% beta with 2.12 MeV maximum energy, and 16% gamma 155 keV) is of current interest for the variety of therapeutic applications. It has a gamma-line at 155 keV (16%) allowing imaging at the normal Tc-99m settings of a gamma-camera. The beta emissions of ^188^Re have a sufficient penetration over a maximum range of 10.8 mm for the tumor ablation, involving pericapsular lesions, while avoiding damage to adjacent nontumorous tissue. Due to its high energy and short physical half-life, ^188^Re offers the possibility of higher energy deposition in a shorter time period relative to radionuclides with longer half-lives. ^188^Re has been used with glass microspheres, human serum albumin microspheres, poly (L-lactide) microspheres, and lipiodol as embolic platforms.^13–17^

Implants of bioactive and biocompatible materials and radioactive sources have been developed in medicine for more than a decade.^18–23^ The first described bioactive material was a glass composed of SiO_2_, CaO, Na_2_O, and P_2_O_5_ by Hench in 1971. The main aspect of bioactive glasses is the reaction control in the physiological environment, commanding the formation of a continuous interface, connecting the tissue with the implanted material. The mechanism that the tissue connects to the implanted material is directly related to the tissue response, the implant interface, and the topography of the material. The tissue response to an inactive material is the formation of a fibrous capsule. The biomaterial's pores allow its fixation to the tissue by the growth of the tissue's cells inside the first's pores. Reabsorbable implants are projected to be gradually degraded in time, being substituted by the local tissue.^24^ The sol-gel process is a chemical technique that can be utilized for producing glasses, ceramics, ceramic glasses, and other composites.^18,25,26^ Here, this technique has been applied to manufacture a new bioactive glass seed. Previous works showed that the high degradability of bioactive glasses can be produced by the sol-gel technique.^6,27–29^ These bioglasses interact with the surrounding tissue in situ and can be absorbed by body after about 7 months.^19,21,30^

The purpose of this study was to illustrate the dosimetric characteristics of the produced ^188^Re glass seed for brachytherapy application. Monte Carlo (MC) dosimetric simulations using MCNP5 code were executed, and radial dose distribution was estimated around the seed. The American association of physicist in medicine (AAPM) task group 43 (TG-43) published a report in 1995 to address the issue of standardizing brachytherapy dosimetry. Although TG-43 deals primarily with gamma-emitting sources, the same procedures have been adopted by AAPM Task Group 60 (TG-60) for brachytherapy and beta dosimetry fields. The recommendations of AAPM Task Group 149 reaffirm the use of TG-43 and TG-60 formalisms.^31–33^ Most importantly, dosimetric factors containing the reference point dose rate, *D* (*r*_0_,*θ*_0_), the radial dose function, *g*(*r*), the 2-dimensional anisotropy function, *F*(*r*,*θ*), the 1-dimensional anisotropy function, φ_an_^(r)^, and the *R*90 amount were computed according to the AAPM TG-60 report recommendations.^31^

## MATERIALS AND METHODS

### The ^188^Re Source Definition

In sol-gel technique, it was possible to produce the ^188^Re bioglass seed. The bioglass seed (0.3 mm diameter and 1.6 mm length) was created inside a Teflon mold, with cylindrical punctures, in order that they could obtain the desired seed format. The sol-gel procedure was initiated, following the steps of gelation, aging, drying, and heat treatment.^28,29^ The seed consisted of a uniform compound comprised of 20% of Rhenium, 50% of silicon, and 30% of calcium. The density of the seed was measured to be 2.35 g/cm^3^. In our research, one sample of bioactive glass seed (Si:Re:Ca) was synthesized and exposed to a thermal neutron flux of 6 to 8 × 10^13^ n/cm^2^/s. Specific activity of the produced ^188^Re from 2 mg of ^187^ReO_2_ was 3 GBq/mg after 18 h of neutron irradiation.

### MC Calculation Performance

MCNP is a general-purpose MC code for simulation of neutrons, photons, and electrons or coupled neutron/photon/electron transport.^34^ The MCNP5 code from Los Alamos National Laboratories was applied to examine the radiation dose distribution in the region of the ^188^Re beta-emitting seed that would be very difficult to investigate experimentally due to the short range of beta particles.

Simulations were executed with the center of the cylindrical seed placed at the midpoint of a 30-cm diameter spherical water phantom allowing for scattering conditions in the region of interest. The radioactive material was supposed to be uniformly distributed in the seed. The outside space of the sphere was void. The tally grid was composed of an array of ring-shaped detectors. These detectors were delineated via the intersection of a series of concentric spherical shells with a series of concentric cones, both originating at the center of the radioactive source. The geometric result was a series of ring-shaped volumes surrounding the source. Detectors as rings, instead of point detectors in a 2-dimensional array, resulted in a much higher probability of tracked photons and electrons going in the defined detector volume. The detectors were simulated at radial distances ranging from 0.3 to 9 mm over angles ranging from 0 to 90 degree in 10-degree increments. The absorbed doses due to electrons and photons were calculated using the MCNP ∗F8 and F6 tallies, respectively. The ∗F8 tally is designed to score the net energy (MeV) deposited in the scoring region, which, when divided by the tally mass (g), yields the raw MC calculated dose rate (MeV/g/source photon) of the scoring region. And also, the F6 tally measures the energy absorbed per gram of material comprising each tally volume.^34^

In these simulations, mcplib04 and el03 cross-section libraries were used.^34^ The ITS 3.0-style energy indexing algorithm was selected over the inherent MCNP-style algorithm through an option in the DBCN card because previously published works have shown that the ITS 3.0-style algorithm gives results closer to those achieved through experimentation.^6,34,35^ Here in this study, the previously published study for a ^142^Pr beta emitter source was applied as a benchmark for validation of our simulation method and dose calculation.^36^

The radiation energy spectra was obtained from the radiological toolbox software package developed at US Nuclear Regulatory Commission.^12^ The complete spectral information for all the particles emitted (beta particles, discrete electrons, gamma rays, and X rays) were input into the MCNP5 simulations by combining the probabilities of emission with their associated energies.

^188^Re emits on average 1.644 particles per disintegration. Of these, 1 is beta particle, 0.376 is discrete electrons, and 0.268 is photons (gamma rays and x-rays). The beta particles and discrete electrons were run together, sampling from their respective distributions. Of the particles chosen at the start of each history, 72.6744% are sampled from the beta spectrum and 27.3256% are sampled from the discrete electron spectrum. Because MCNP5 does not permit a mixed photon and electron source, 2 separate calculations were performed and the total dose was estimated from the sum of these 2 components. According to this point of view, we have chosen to express results in units of cGy/h/μCi. The dose in units of MeV/g per particle was obtained using the outputs in the tallying cells for the following 2 cases: betas and discrete electrons, and photons. The dose rate for betas and discrete electrons was converted to cGy/h/μCi by the following approach, which was employed with the AAPM Task Group 60 dose formalism: 
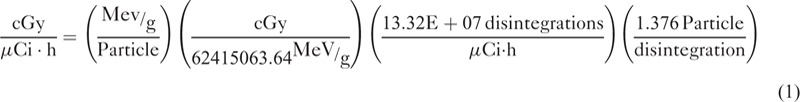


In the same way, for photons, the dose rate was calculated by considering the number of photons emitted per disintegration. The number of simulated beta particle histories was 5 × 10^8^ for the source. This amount of histories produced statistical uncertainties of 0.5% and 1.6% at 2 mm and 7 mm on the transverse plane, and 0.8% and 4% at 3 mm and 7 mm along the long axis, correspondingly. The number of photon histories was set at 3 × 10^8^ for the simulation to obtain a relative statistical error in the simulation not >0.5% at all distances.

### The AAPM Task Group 60 Factors

The AAPM Task Group 60 endorsed that in place of air kerma strength and a dose rate constant, the reference dose rate should be used for calculation of dose distribution around a beta emitter source. With reference to this protocol, the dose calculation at a point (*r*,*θ*) is determined using the following formalism:^31^ 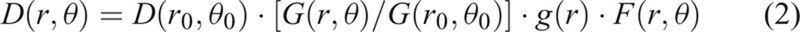


where *D*(*r*_0_,*θ*_0_) is the dose rate in water at the reference point (*r*_0_,*θ*_0_), *G*(*r*,*θ*) is the geometry function, *g*(*r*) is the radial dose function, and *F*(*r*,*θ*) is the 2D anisotropy function. The reference point suggested in AAPM TG-60 for beta sources is *r*_*0*_ = 2 mm and *θ*_*0*_ = 90 degree.

The geometry function, G(*r,θ*), was computed for a simple line source with the same effective length of the seed via the F4 fluence tally (1/cm^2^) in each detector in a vacuum external the source.^37^ The 2D anisotropy function, *F*(*r*,*θ*), characterized the angular dependence of the dose rate in the region of the source, and comprises the impacts of scattering and absorption inside water or tissue. The 2D anisotropy function of the source was computed inside water at 10 degree intervals at radial distances ranging from 0.3 to 8 mm from the source center via MC simulations.

The *g*_*L*_(*r*) as radial dose function explains for radial dependence of beta absorption and scatter in water or tissue along the transverse axis.^31^ The radial dose function values of the ^188^Re seed were calculated in Water at radial distances ranging from 0.3 to 8 mm.

To guarantee that the presented TG-60 factors are accurate, the dose rate and the radial dose function, *g*(*r*), amounts intended for a ^142^Pr beta-emitting source, which was previously studied.^35^

The *R*90 quantity shows the distance from the point source within which 90% of the energy is absorbed inside a medium. The *R*90 amount was computed by modeling a point source in the center of a series of concentric spherical shells with a thickness of 0.01 mm at radial distances ranging from 0.01 to 15 mm inside a water phantom.

## RESULTS AND DISCUSSION

The short range of the beta particles causes a more rapid dose reduction versus distance than the gamma sources. Beta-emitting sources submit the advantage of allowing treatment of tissue adjacent to the source without delivering major dose to close sensitive structures. And also, these sources are easy to shield and can be achieved with the great specific activity to provide for short treatments, which can be put in order even in rooms that are not heavily shielded.

### The Dose Rate Profiles

Table 1 demonstrates the comparisons between the MC calculated amounts of the present work and the published data. The differences between dose rate amounts at distances from 1 to 7 mm were ranging from 0.07% to 4.55%. Meanwhile, in the Table 1 the differences between two studies were found to be less than 1.2%. These comparisons reveal good agreement.

**FIGURE 1:**
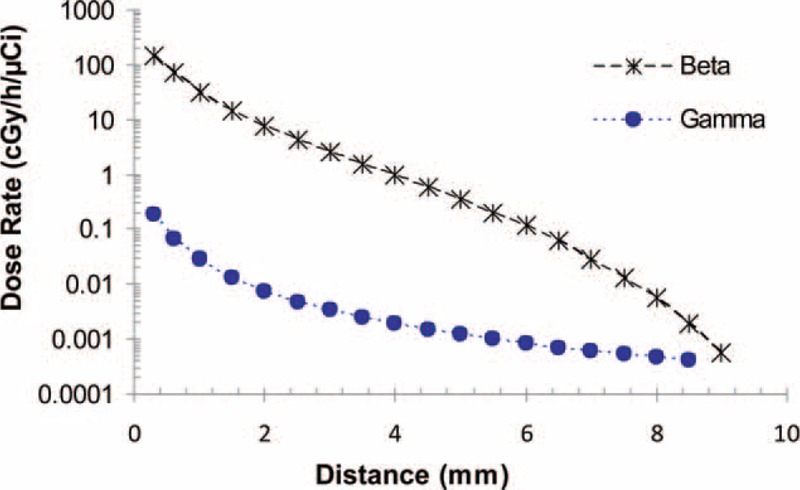
The Monte Carlo calculated radial dose rate profiles of beta and gamma radiation of the ^188^Re seed.

The MC calculated dose rate amounts for beta and gamma radiation concerning the ^188^Re seed center are arranged in Table 2. The dose rate at the reference dose point means *D*(*r*_0_,*θ*_0_), for the ^188^Re seed was determined to be 7.43 ± 0.5 cGy/h/μCi. Based on the pervious works on the other beta emitting sources, the value for ^142^Pr, ^90^Sr/^90^Y and ^32^P was computed to be 3.3336, 2.412, and 6.0912 cGy/h/μCi, respectively.^7,36,38^Figure 1 depicts the dose rate distributions from beta and gamma radiation of the ^188^Re seed inside the water. Therefore, Figure 1 confirms the small amount of gamma contribution to the total dose distribution. Two-dimensional dose rates per unit included activity (cGy/h/μCi) were computed for the ^188^Re seed from 0.3 to 8 mm via the MC code and are demonstrated in Table 3.

**TABLE 2:**
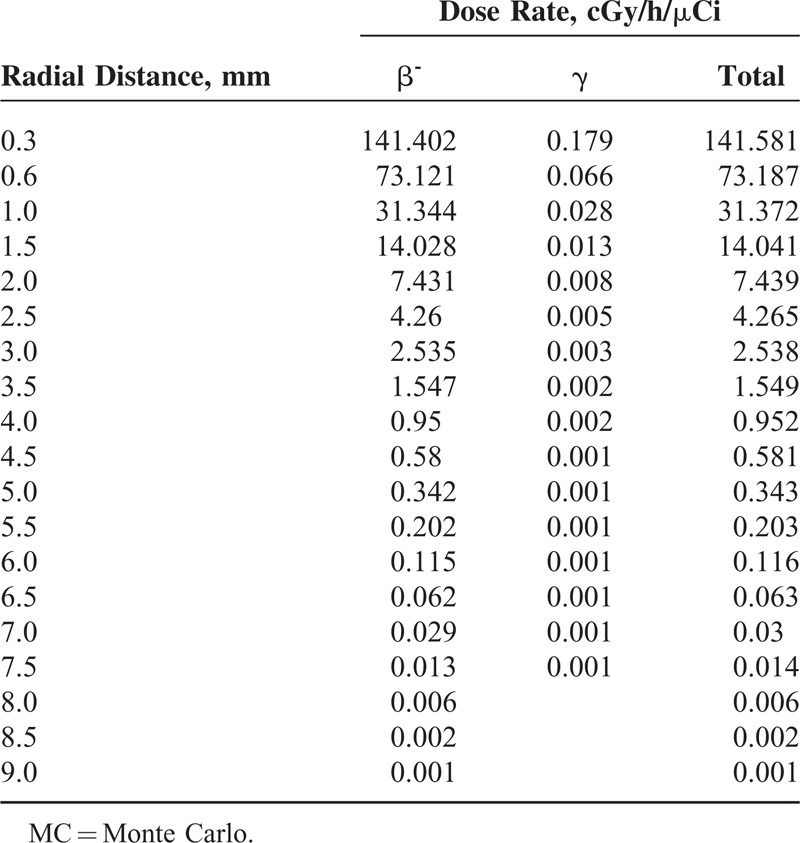
The MC Calculated Dose Rate Amounts for Beta and Gamma Radiation of the ^188^Re Seed

**TABLE 3:**
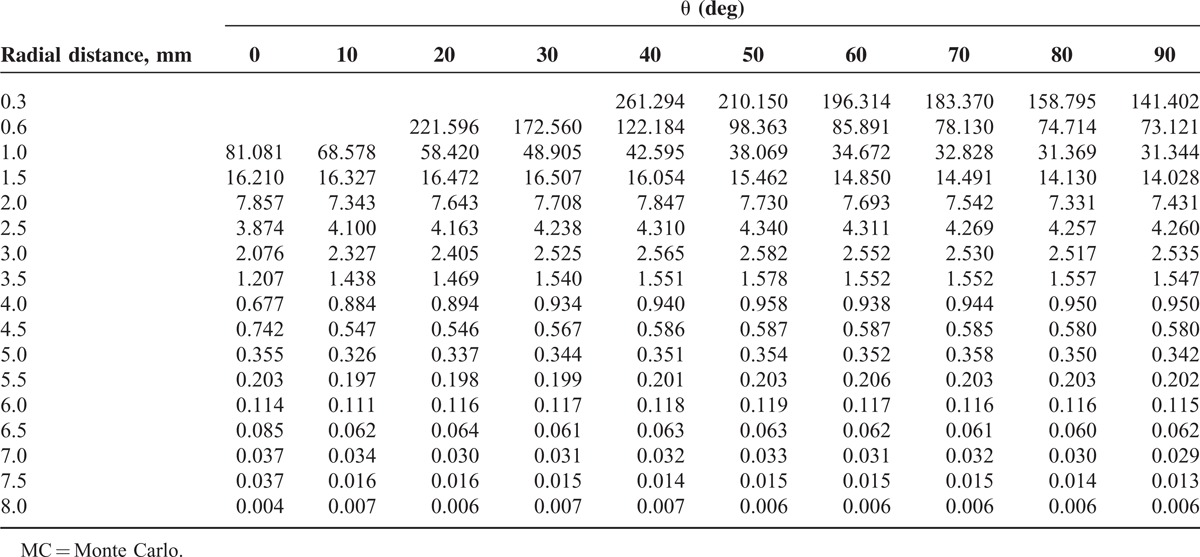
The MC Calculated 2-Dimensional Dose Rate (cGy/h/μCi) for the ^188^Re Seed

**FIGURE 2:**
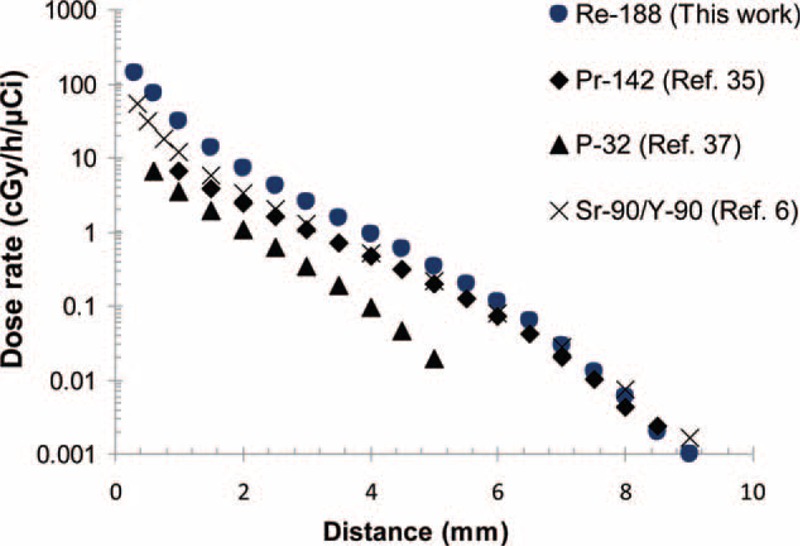
Comparison of the radial dose rate profile of the ^188^Re seed with various attainable beta emitter sources.

Figure 2 shows a comparison of the radial dose profile of the ^188^Re seed with other beta emitter sources (Pr-142 (Ref. 35), P-32 (Ref. 37), Sr-90/Y-90 (Ref. 6)) as a function of radial distance along the transverse axis. It can be founded that the dose profile of ^188^Re is in a logical range compared with those of other beta emitter seeds. As expected for a beta-emitting, a great radial dose gradient exists in the near-source region inside water. This is one characteristic of the beta sources, which deposit more energy into a limited region adjacent to the source while the electron velocity decreases. Remarkably, it is possible to irradiate small target volumes with a high dose rate by a set of radioactive seeds, minimizing the damage in healthy nearby tissues.

The radial dose amounts of the ^188^Re seed were higher than the other seeds at radial distances <5 mm. The dose profile reduced sharply to 2 mm due to low-energy part of the spectrum of beta particles and dropped more quickly than the dose profiles of the other seeds after 5 mm. The dose profiles are reliant upon the seeds’ dimensions and the range of the beta particles. Because of the short range of beta particles, the dose to close organs can be minimized with optimized implantation locations of the seeds.

### Assessment of g_L_(r) Function as Radial Dose Factor

The computed numerical amounts of the radial dose function for the ^188^Re seed have presented in Table 4. The radial dose function inside water for distances from 0.03 to 0.8 cm was fitted to a 5^th^ order polynomial function as follows: 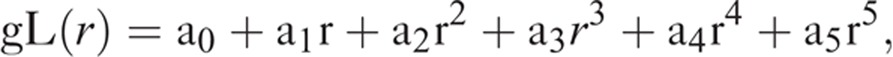


**TABLE 4:**
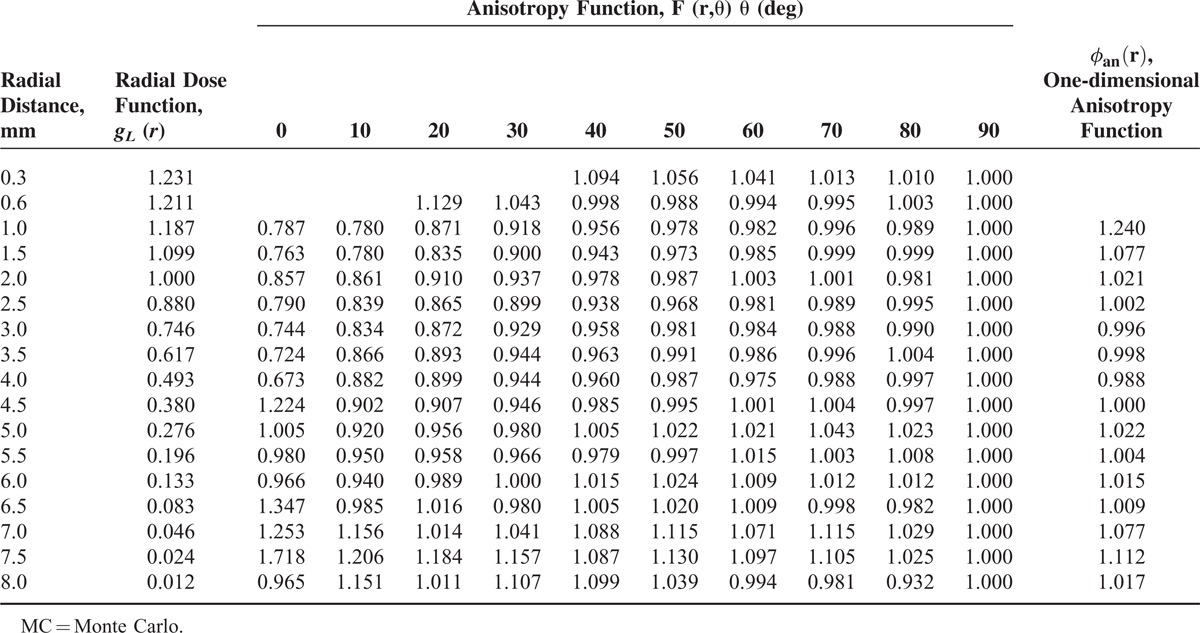
The MC Calculated Radial Dose function, *g*_*L*_(*r*), and Anisotropy Function, *F*(*r*,*θ*), for the ^188^Re Seed

Where *a*_0_ = 1.21747, *a*_1_ = 0.068, *a*_2_ = −0.12046, *a*_3_ = 0.01698, *a*_4_ = −5.47722E-4, and *a*_5_ = −1.49945E-5, define *R* = 0.99993.

**FIGURE 3:**
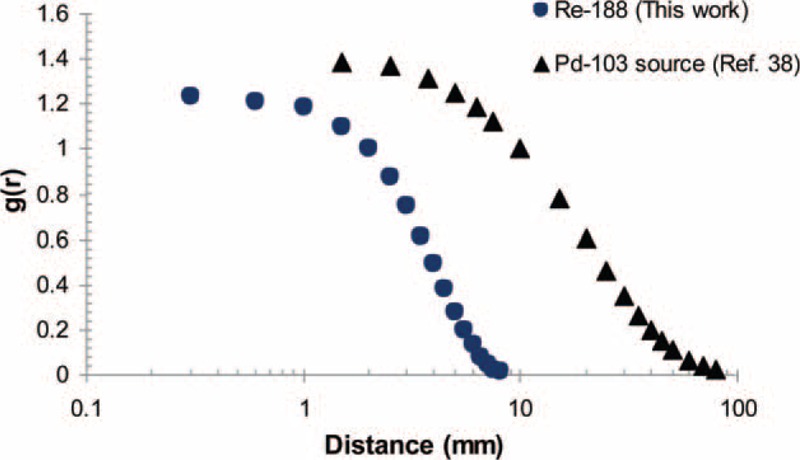
Comparison of the Monte Carlo calculated radial dose functions of the ^188^Re and the IRA-^103^Pd seed.

**FIGURE 4:**
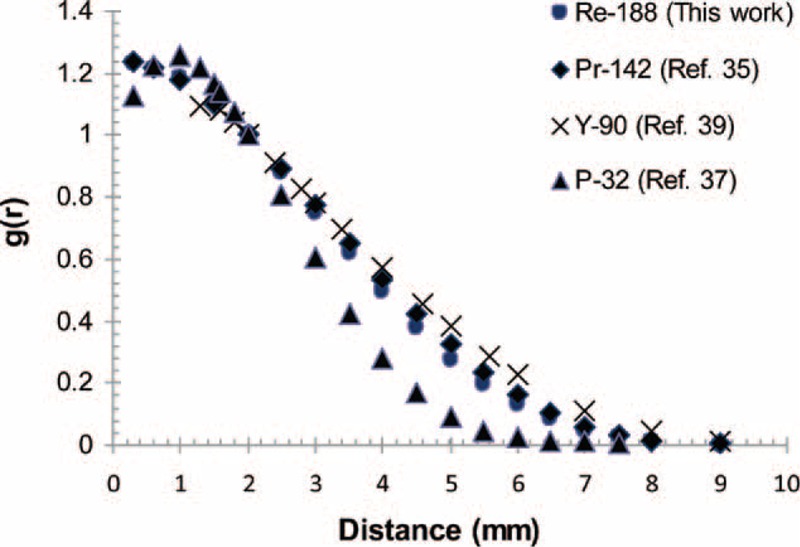
Comparison of the Monte Carlo calculated radial dose function of the ^188^Re seed with those of the ^142^Pr, ^90^Y, and ^32^P sources.

Figure 3 be evidence for the comparison of the radial dose functions of ^188^Re and IRA-^103^Pd seeds.^39^ The radial dose function value for IRA-^103^Pd reduces slowly in distances >10 mm, whereas the radial dose function profile for ^188^Re falls off quickly at about 2 mm, and then the absorbed dose by the healthy adjacent organs is diminished. The radial dose function of the ^188^Re seed and the other available beta emitter sources has shown graphically in Figure 4. The dose amounts for the ^188^Re seed decline like the ^90^Y and ^142^Pr sources.^36,38,40^

### The F(r,θ) Appraisal, the 2D Anisotropy Function

**FIGURE 5:**
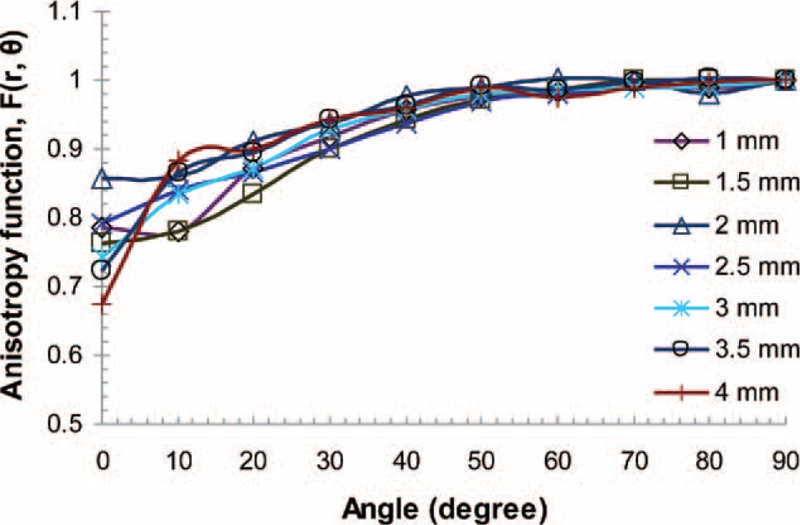
The Monte Carlo calculated 2D anisotropy function of the ^188^Re source at selected radial distances.

On the basis of 2-dimensional dose rate distribution presented in Table 3, the anisotropy function for the source was derived. Table 4 presents the *F*(*r*,*θ*) amounts. The function is shown graphically in different radial distances in Figure 5. The φ_an_^(r)^, one-dimensional anisotropy function was calculated from the values in Table 4 by averaging over all solid angles as described in the TG-43 report. The parameter amounts given in Table 4 were found to be close to unity nearly at all radii.

### The *R90* Amount and the Isodose Curves

**FIGURE 6:**
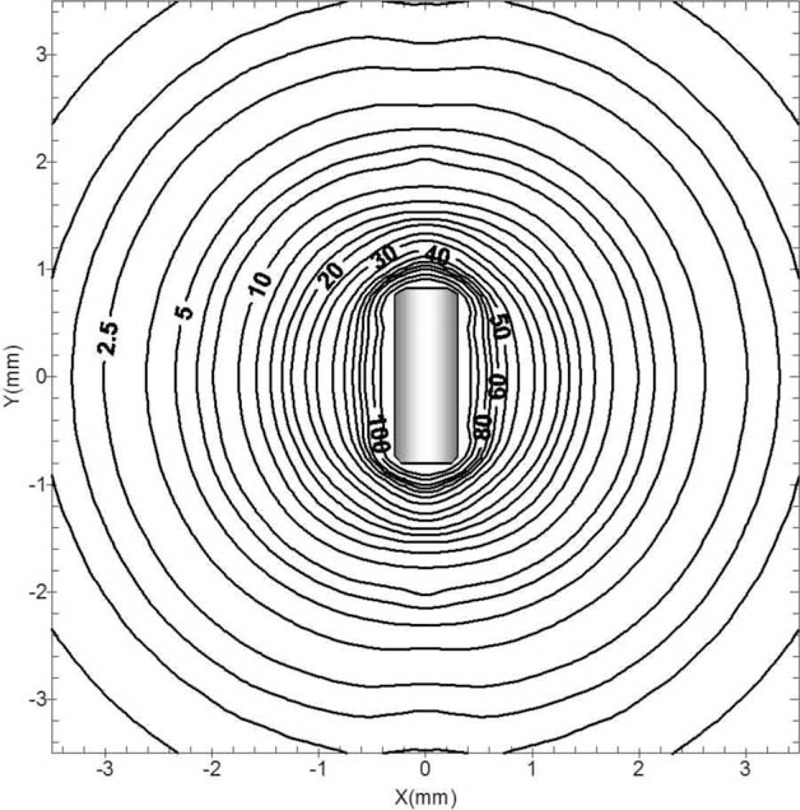
Isodose rate curves of the ^188^Re source. Isodose lines are presented in units of cGy/h/μCi.

The *R*90 amount parameter for ^188^Re was computed to be 0.48 mm. Isodose rate contours around the source were plotted in Figure 6, covering the region up to 3.5 mm from the source center in the longitudinal path. It is clear from the isodose curves that the dose rate tumble is rapid in the radial track. Dose distribution of this seed produces to be greater isotropic than other commercially attainable encapsulated seeds, as it has no end weld to attenuate radiation.

## CONCLUSIONS

A beta-emitting ^188^Re bioglass seed was examined as a potential candidate for treating liver cancer. MC dosimetric calculations by MCNP5 code were executed inside water and the dosimetric factors defined in AAPM TG-60 were estimated for the seed containing: the reference dose rate, the radial dose function, and the anisotropy function. By knowing the seed specifications such as the energy generated per seed and the seed dosimetric factors, the number of the seeds needed for the elimination of the tumor volume can be predicted and a suitable treatment planning for brachytherapy applications can be performed.

Although the ^188^Re seed degradation and its limited range of beta particles are good benefits, however, further studies are required to explore the potential of this seed.
